# Egg ovotransferrin‐derived ACE inhibitory peptide IRW increases ACE2 but decreases proinflammatory genes expression in mesenteric artery of spontaneously hypertensive rats

**DOI:** 10.1002/mnfr.201500050

**Published:** 2015-06-26

**Authors:** Kaustav Majumder, Guanxiang Liang, Yanhong Chen, LeLuo Guan, Sandra T. Davidge, Jianping Wu

**Affiliations:** ^1^ Department of Agricultural, Food and Nutritional ScienceFaculty of Agricultural, Life and Environmental Sciences, University of AlbertaEdmontonABCanada; ^2^ Cardiovascular Research Centre, University of AlbertaEdmontonABCanada; ^3^ Women and Children's Health Research Institute, University of AlbertaEdmontonABCanada; ^4^ Department of Obstetrics and GynecologyFaculty of Medicine and DentistryUniversity of AlbertaEdmontonABCanada

**Keywords:** ACE‐2, Bioactive peptides, Hypertension, RNAseq, SHR

## Abstract

**Scope:**

Egg ovotransferrin‐derived angiotensin converting enzyme (ACE) inhibitory peptide IRW was previously shown to reduce blood pressure in spontaneously hypertensive rats through reduced vascular inflammation and increased nitric oxide‐mediated vasorelaxation. The main objective of the present study was to investigate the molecular mechanism of this peptide through transcriptome analysis by RNAseq technique.

**Methods and results:**

Total RNA was extracted from kidney and mesenteric arteries; the RNAseq libraries (from untreated and IRW‐treated groups) were constructed and subjected to sequence using HiSeq 2000 system (Illumina) system. A total of 12 764 and 13 352 genes were detected in kidney and mesenteric arteries, respectively. The differentially expressed (DE) genes between untreated and IRW‐treated groups were identified and the functional analysis through ingenuity pathway analysis revealed a greater role of DE genes identified from mesenteric arteries than that of kidney in modulating various cardiovascular functions. Subsequent qPCR analysis further confirmed that IRW significantly increased the expression of ACE‐2, ABCB‐1, IRF‐8, and CDH‐1 while significantly decreased the expression ICAM‐1 and VCAM‐1 in mesenteric arteries.

**Conclusion:**

Our research showed for the first time that ACE inhibitory peptide IRW could contribute to its antihypertensive activity through increased ACE2 and decreased proinflammatory genes expression.

**Abbreviations**
ACE: angiotensin converting enzyme
CVD: cardiovascular disease
CPM: counts per million
DE: differentially expressed
eNOS: endothelial nitric oxide synthase
FC: fold change
IPA: Ingenuity Pathway Analysis
PPAR: peroxisome proliferator activator receptor
RNAseq: RNA‐sequencing
RAS: renin angiotensin system
SHR: spontaneously hypertensive rat

## 1 Introduction

Hypertension, the persistent increase of systolic/diastolic blood pressure over 140/90 mm Hg, respectively, is a major health concern afflicting approximately 1 billion people worldwide 1. Hypertension is considered as one of the risk factors for cardiovascular diseases (CVDs) accounting for ∼30% of global deaths 2, 3. Since pharmacological antihypertensive therapies are often associated with adverse side effects 4, 5, a safer therapeutic alternative is highly desirable; due to their natural origins and perceived lack of adverse side effects, food protein derived bioactive peptides are considered as these alternatives 10, 11. Blood pressure is regulated through multiple mechanisms; these food‐derived peptides were also reported to reduce blood pressure through multiple pathways, i.e. inhibition of angiotensin converting enzyme (ACE) and renin, increased expression of endothelial nitric oxide synthase, and concomitant release of nitric oxide 6, 7, 8, 9.

A novel ACE inhibitory peptide, IRW, identified from egg white protein ovotransferrin 15, was also shown to have anti‐inflammatory effects via modulated expression of several adhesion molecules and inflammatory cytokines in cells 17. Our further in vivo study in spontaneously hypertensive rats (SHRs), a widely used animal model of essential hypertension, demonstrated pronounced antihypertensive effects of orally administered IRW that were likely mediated through several different pathways such as modulation of renin angiotensin system (RAS) through ACE inhibition, reduced vascular inflammation, increased nitric oxide (NO) mediated vasorelaxation though increased expression of endothelial nitric oxide synthase (eNOS) and NO bioavailability by reducing oxidative/nitrosative stress 18. Similarly, two well known, milk‐derived antihypertensive peptides IPP and VPP were also reported to have anti‐inflammatory effects in vascular tissue of apolipoprotein‐E knockout mice and adipose tissue of mice fed with high‐fat diet 19, 20. Lactoferrin‐derived peptide RPYL was reported to inhibit angiotensin II (Ang II) induced vasoconstriction by blocking the binding of Ang II with angiotensin receptor 1 (AT_1_) in rabbit carotid artery 21. Therefore the mechanism of actions of ACE inhibitory peptides was far more complicated than the inhibition of ACE; it is likely that many of these bioactive peptides exert health benefits through modulation of various biologically active proteins at the transcriptional and posttranscriptional levels. Therefore it is important to evaluate the functions of the bioactive peptides on the transcriptome level to identify the alteration of gene expression that may be responsible for the biological effect.

The transcriptome is defined as the complete set of RNA produced by the genome at a given moment in a given tissue in a selected organism; it is considered an important link between the phenotype and the information encoded in the genome 25. A high throughput technique termed RNA‐sequencing (RNAseq) is now available for genome‐wide profiling of the entire transcriptome 24, 25, which can detect the lowly expressed transcripts through in depth sequencing 26. RNAseq provides opportunities to explore many different aspects of the entire transcriptome and is also useful to quantify the gene expression levels by digital analysis 24. Thus this technique can be applied to investigate the detailed molecular mechanisms of food‐derived compounds. Therefore, the main objective of the present study was to investigate the molecular mechanism of egg protein ovotransferrin derived ACE inhibitory peptide IRW on SHRs through transcriptome analysis by RNAseq technique.

## 2 Materials and methods

### 2.1 Animal experimental procedures and sample collections

Animal experiments were performed in SHR as previously described 18. Animals were randomly assigned to treatment groups: untreated (control, *n* = 6), and IRW (15 mg/Kg BW, *n* = 6) for a period of 18 days, and then were sacrificed, and tissues (kidney, aorta, heart, mesenteric arteries; *n* = 6) were collected, weighed, and stored in RNA stabilization reagent (RNA*later*, Qiagen, Toronto, Ontario) at –80°C for future experiments. All experimental protocols were reviewed and approved by the University of Alberta Animal Welfare Committee (Protocol # 611/09/10/D) in accordance with the guidelines issued by the Canada Council on Animal Care and also adhered to the Guide for the Care and Use of Laboratory Animals published by the United States National Institutes of Health. Mesenteric arteries were dissected from the anterior portion of the abdominal aorta and cleaned of adherent surrounding adipose and connective tissues in sterile ice‐cold HEPES‐PSS (in mmol/L: NaCl 142, KCl 4.7, MgSO_4_ 1.17, CaCl_2_ 4.7, K_2_PO_4_ 1.18, HEPES 10, and glucose 5.5; pH 7.4) solution in a petri dish. The mesenteric arteries were put in RNA stabilization reagent after cleaning, whereas kidneys were directly put into RNA stabilization reagent and stored in –80°C.

### 2.2 RNA isolation

Kidney tissue samples were ground into fine powder in liquid nitrogen using oven baked (for sterilization), prechilled mortar and pestle prior to nucleic acid extraction, whereas mesenteric artery samples were cut into small pieces using RNA*later* RNA Stabilization Reagent (Qiagen) by sterile surgical blades. The tissue samples (∼80 mg) were first homogenized using a Precellys1 24 homogenizer (Bertin Technologies, Montigny, France) with two cycles of 30 s each at 5500 rpm and 10 s pause in between and the total RNA were then extracted using *mir*Vana^TM^ miRNA Isolation Kit (Ambion, Carlsbad, CA), in a phenol‐free manner according to the manufacturer's instructions. The quality and quantity of the RNA were determined using Agilent 2100 Bioanalyzer (Agilent Technologies, Santa Clara, CA) and Qubit 2.0 Fluorometer (Invitrogen, Carlsbad, CA), respectively. Only the RNA samples with integrity number (RIN) higher than or equal to 7.0 were preceded for RNA‐seq library construction.

### 2.3 RNA‐seq library construction and sequencing

Total RNA (20 ng/μL) from each sample was used to construct RNA‐seq libraries with a unique index using the TruSeq mRNA Sample Preparation kit (Illumina, San Diego, CA) and the quantification of the library was performed using Qubit 2.0 Fluorometer (Invitrogen, Carlsbad, CA). The libraries were sent to Génome Québec (Montréal, Canada) for sequencing on a HiSeq 2000 system (Illumina). Sequencing reads were 100 bp paired‐end, and they were demultiplexed according to their index numbers with CASAVA version 1.8 (Illumina). Reads that did not pass the Illumina chastity filter test were removed for further analysis.

### 2.4 RNA‐seq reads mapping and annotation

Rat genome (*Rattus norvegicus*, Rnor_5.0) was downloaded from ENSEMBL (http://uswest.ensembl.org/) server and RNA‐seq reads were aligned to the rat genome by using Tophat 2.0.10 with default parameters 27. The output mapping files from the TopHat2 alignment, along with the GTF file from ENSEMBL (http://uswest.ensembl.org/) rat gene annotation v75.30, were used in the htseq‐count (http://www.huber.embl.de/users/anders/HTSeq/) to determine the total number of reads mapped to each gene. The abundance of mRNAs in each library was obtained by normalizing reads number to counts per million reads (CPM), calculated as (gene reads number/total mapped reads number per library) × 1 000 000. The expressed genes with CPM > 1 in at least 60% of the samples was used for further analysis.

### 2.5 Identification of differentially expressed genes

Differential expression of genes between untreated (Untrt), and IRW‐treated (IRW) groups were then assessed using edgeR bioinformatics tool 28. Only higher abundance genes (CPM>5 in at least in 60% of the samples) were used for the identification of differentially expressed (DE) genes. The significantly DE genes were determined based on Benjamini and Hochberg multiple testing correction test by calculating the false discovery rate < 0.05 and fold change (FC) >1.5 (log_2_FC > 0.6) 29, which is similar to a previous study by Liang et al. 30.

### 2.6 Functional analysis of differentially expressed genes

Ingenuity pathway analysis (IPA, Ingenuity Systems, www.ingenuity.com) was used to analyze the correlation between gene expression and key functions of vascular tissues and kidney. A threshold of value of *p* < 0.01 was applied to enrich significant biological functions. The functions of significantly expressed DE genes from both the tissues were analyzed. The functional change according to the FC of DE genes and the direction for a given function (increase or decrease) were determined by using the *z*‐score algorithm provided by the IPA server.

### 2.7 Quantitative real‐time PCR (qRT‐PCR) analysis

To verify the DE genes obtained from RNA‐seq analysis, qRT‐PCR was performed on selected genes: angiotensin converting enzyme 2 (ACE‐2), ATP‐binding cassette‐subfamily B (ABCB‐1), interferon regulatory factor 8 (IRF‐8), E‐Cadherin (CDH‐1), intercellular adhesion molecule 1 (ICAM‐1), and vascular cell adhesion molecule‐1 (VCAM‐1). The gene‐specific primer pairs were designed using Primer Express software (Applied Biosystems, Foster City, CA) (Table 1). The total RNA was reversely transcripted to synthesize the first strand and second strand of complementary DNA (cDNA) using SuperScript® II Reverse Transcriptase (Invitrogen by Life Technologies, Foster City, CA, USA). The qRT‐PCR was performed with Step OnePlus^TM^ Real‐Time PCR System (Applied Biosystems by Life Technologies, Foster City, CA, USA) using the SYBR green chemistry. Thermal cycling was conditioned at 95°C for 20 s for initial denaturation, and then 40 cycled at 95°C for 3 s followed by annealing/extension for 30 s at specific temperature for each primer pair (Supporting Information Table 1). The expression levels were calculated at the cycle threshold values (*C*_T_), where the fluorescent signal was detected above background intensity. The relative gene expression was calculated by using ß‐actin as a reference gene. Relative gene expression was calculated based on ΔCT and ΔΔCT values, where ΔCT = [CT (Target gene) – CT (β‐actin)]; and ΔΔCT = [ΔCT (IRW) – ΔCT (Untrt)]. The fold changes between the untreated (Untrt) and treated (IRW) groups were obtained using 2^−ΔΔCT^.

**Table 1:** Effect of IRW treatment on differentially expressed (DE) genes from the mesenteric artery (MA) and their biological functions

Gene name	Type	Effects on cardiovascular function	Fold change in RNAseq	Fold change in qPCR
Angiotensin converting enzyme‐2 (ACE‐2)	Enzyme	Vasoconstriction Hyperactivity of renin angiotensin system	+24‐fold	+18‐fold
ATP‐binding cassette, subfamily B‐1 (ABCB‐1)	Transporter	Vasoconstriction Leukocyte quantity	+15‐fold	+158‐fold
Interferon regulatory factor‐8 (IRF‐8)	Transcription regulator	Inhibit hyperplasia	+9‐fold	+7‐fold
E‐cadherin (CDH‐1)	Transporter	Inhibit hyperplasia	+19‐fold	+45‐fold
Intercellular adhesion molecule 1 (ICAM‐1)	Transmembrane receptor	Inhibit vascular inflammation	−3‐fold	−8‐fold
Vascular cell adhesion molecule ‐1 (VCAM‐1)	Transmembrane receptor	Inhibit vascular inflammation	−3‐fold	−7‐fold

Fold change after RNAseq analysis was performed by calculating logfc (logfc = log_2_treatment/untreated). Upregulation of gene expression is expressed by “+” sign and downregulation by “=” sign. Fold change for RNAseq = 2^∧^log_2_fc; fold change for qPCR = 2^−ΔΔCT^, ΔΔCT = [ΔCT (treatment) − ΔCT (untreated)].

The qRT‐PCR data were analyzed using GraphPad Prism 5 (GraphPad software, San Diego, CA) and subjected to unpaired, two‐tailed *t*‐test (*n* = 5–6/group) to determine the significant difference, if any, between Untrt and IRW groups. A *p* value of <0.05 was considered statistically significant.

### 2.8 Data submission

All the RNA‐seq data were deposited in the publicly available NCBI's Gene Expression Omnibus Database (http://www.ncbi.nlm.nih.gov/geo/). The data are accessible through GEO Series accession number GSE65017.

Web link: http://www.ncbi.nlm.nih.gov/geo/query/acc.cgi?acc=GSE65017.

## 3 Results

### 3.1 Overview of RNA‐seq dataset

**Figure 1:**
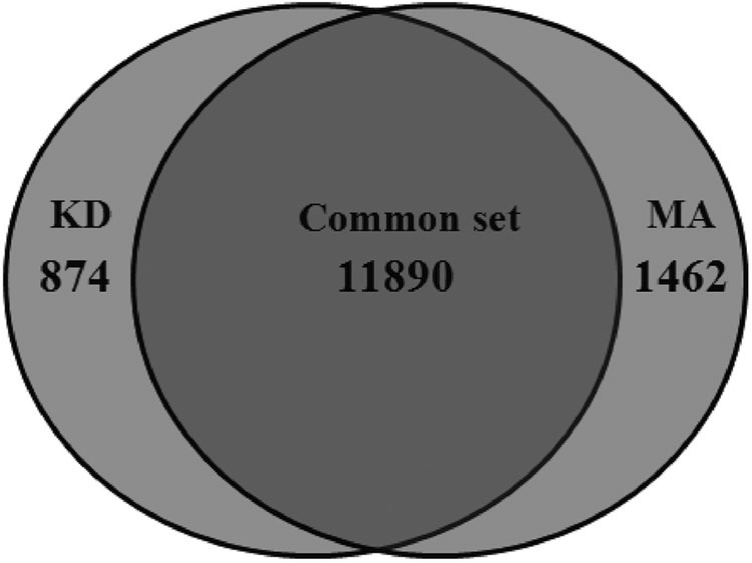
Comparison of gene number detected in kidney (KD) and mesenteric arteries (MA). Total 12 764 genes and 13 352 genes were detected in KD and MA, respectively; out of the 11 890 genes are common to both the tissues types, 874 genes were exclusive to KD and 1462 genes were exclusive for MA.

A total of 658 605 105 high quality 100 bp reads were obtained from 23 libraries (12 samples from kidney, six Untrt and six IRW, and 11 samples from mesenteric artery, five Untrt and six IRW) with an average of 28 M ± 6 M reads per sample. About 82% of reads from kidney and ∼87% in mesenteric artery were mapped to the rat genome (*Rattus norvegicus*, Rnor_5.0). In total, 12 764 expressed genes were detected (CPM>1 in at least 60% of the samples) in kidney and 13 352 genes in the mesenteric artery. Among these genes, 11 890 genes were expressed in both kidney and mesenteric artery, while 874 genes were exclusive to kidney and 1462 genes to mesenteric artery (Fig. 1).

### 3.2 Significantly altered gene expression profiles in response to IRW

**Figure 2:**
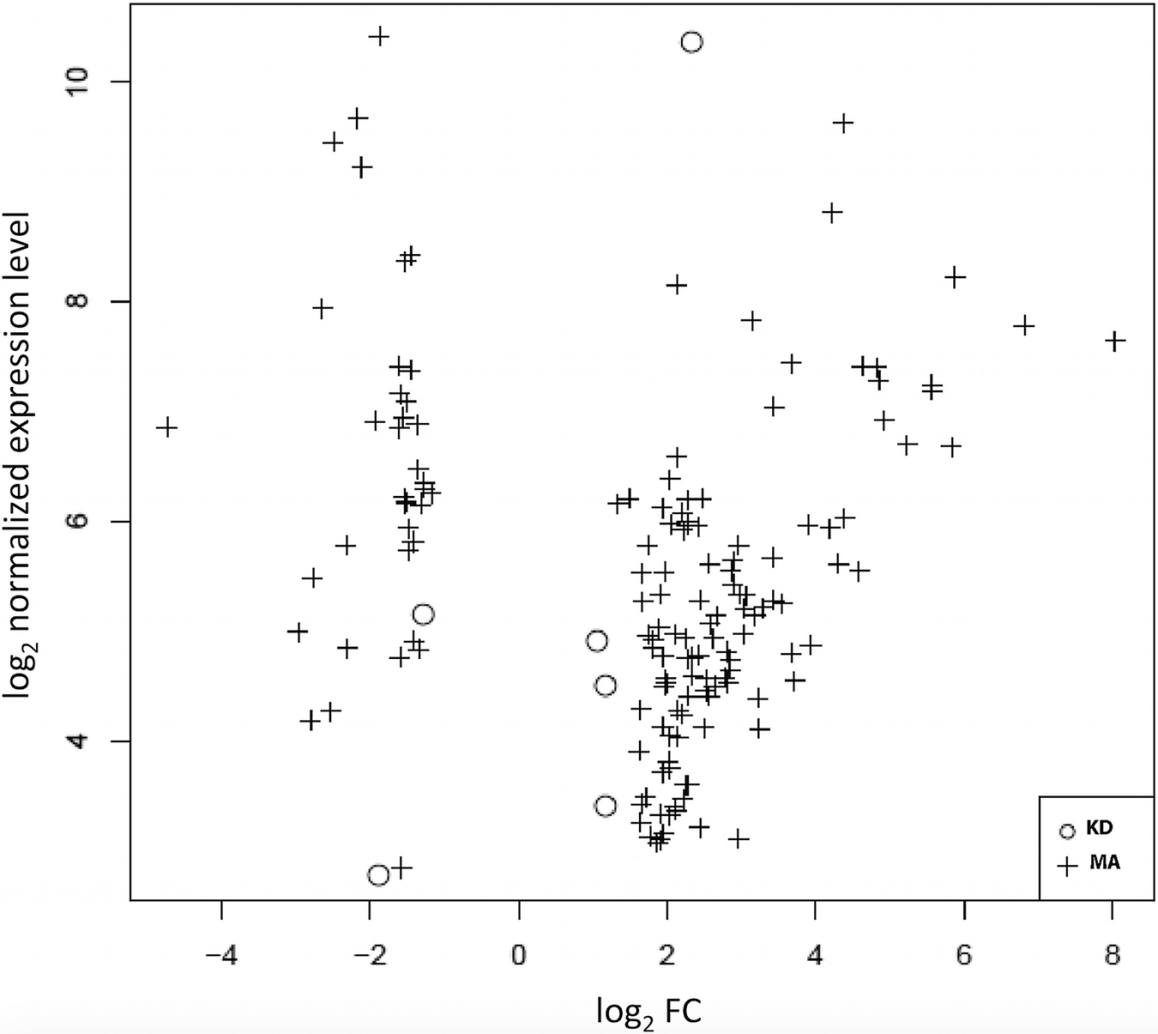
Effect of IRW on detected differentially expressed (DE) genes in kidney (KD) and mesenteric arteries (MA). Axis *X* and *Y* show log2 (fold change) and log2 (normalized reads number) of each DE genes, respectively. DE genes detected in the KD represent by symbol “O” and DE genes detected in MA represent by symbol “+.” The fold changes of the DE genes from KD are much less than gene detected from MA.

Analysis revealed that only 17 genes were DE in kidney, two of them were downregulated and the rest were upregulated after IRW treatment. In comparison, 151 genes were DE in mesenteric artery, out of those 37 genes were downregulated and 114 genes were upregulated. All the DE genes identified in both tissues are listed in the Supporting Information Table 2. DE genes were obviously more pronounced in mesenteric artery than that of kidney in terms of fold change after IRW treatment (Fig. 2).

### 3.3 Functions of DE genes in response to IRW

**Figure 3:**
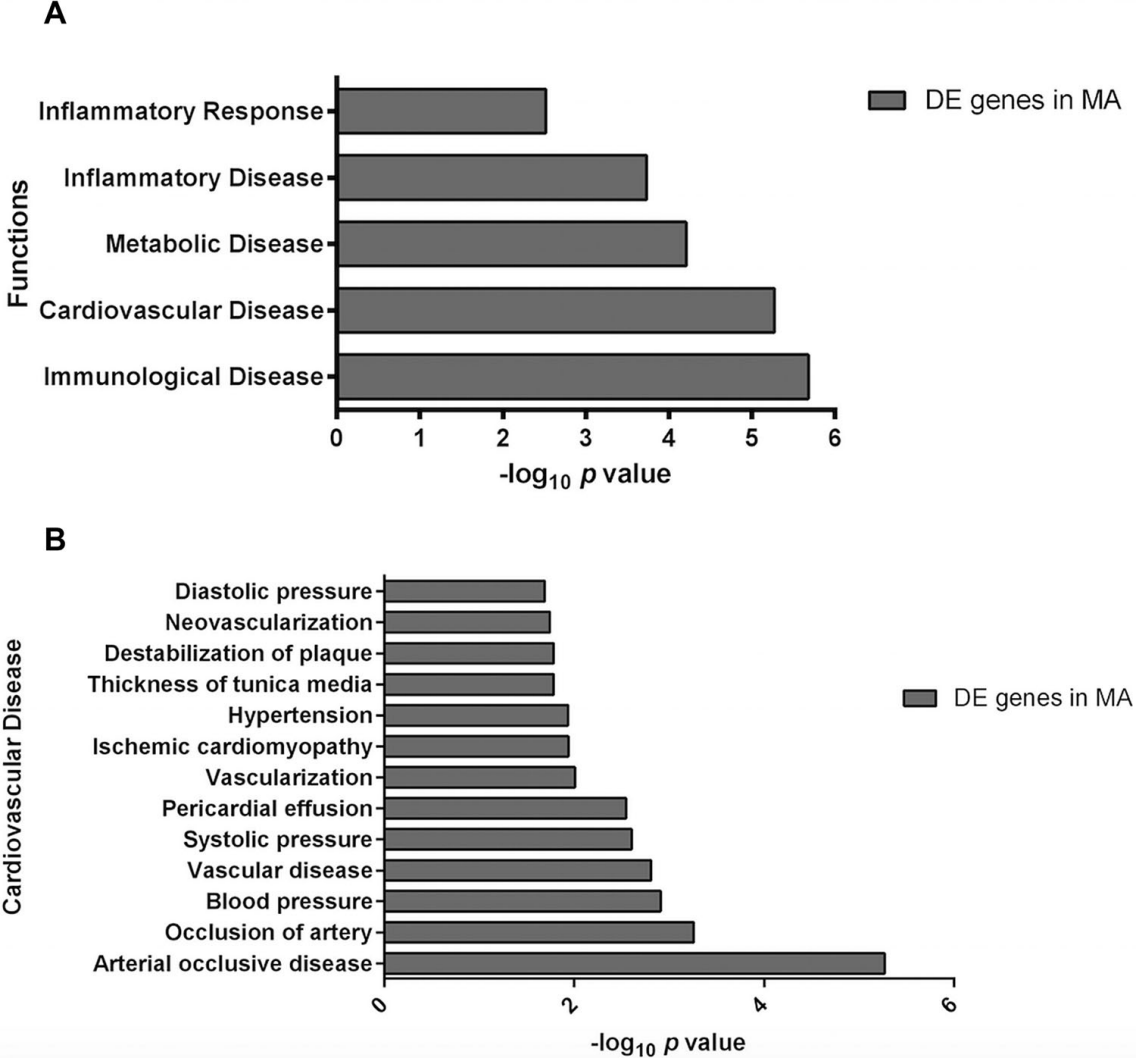
Functional role of DE genes from mesenteric arteries (MA). DE genes identified in the MA after IRW treatment can modulate various physiological functions (A): *X*‐axis and *Y*‐axis represent –log_10_ *P* value and functions, respectively. A higher –log_10_ *P* value indicates a greater relevance to that function. The impact of DE genes identified from MA on cardiovascular disease (B). *X*‐axis and *Y*‐axis represent –log_10_ *P* value and cardiovascular disease functions, respectively. Results were obtained from IPA analyses from *n* = 5–6 per treatment for mesenteric arteries.

Functional analysis conducted through IPA server revealed that DE genes after IRW treatment could modulate various physiological functions. As the focus of the current study was to evaluate the effect of IRW treatment on hypertension, further analysis was performed to delineate the functions of the DE genes that can modulate various cardiovascular functions. The DE genes found in the kidney have a modest effect in cellular functions and not altered any pathways related to the cardiovascular system whereas DE genes from the mesenteric artery have significantly affect the functions such as cellular movement, cell‐to‐cell signaling/interaction, molecular transport, cellular morphology, and cellular maintenance. These altered cellular and molecular functions in mesenteric artery can modulate various diseases and disorders such as inflammatory disease, inflammatory response, metabolic disease, cardiovascular disease, and immunological function (a higher ‐log_10_ *P* value indicates the greater relevance of the DE genes to that particular function, Fig. 3A). The result indicates that IRW treatment can modulate the expression of genes that are highly related to the cardiovascular function. Further analysis showed that the DE genes from mesenteric arteries could modulate various cardiovascular disease functions such as occlusion of artery, hypertension, and ischemic cardiomyopathy (Fig. 3B). Interestingly, DE genes that can modulate cardiovascular function also play important roles in controlling inflammation, immunological and metabolic functions. A total of 32 genes were affected in mesenteric arteries that can modulate these various cardiovascular functions, a detailed list of these DE genes and their respective functions in vascular tissues are presented in the Supporting Information Table 3. Given these results, IRW treatment had a greater effect on the mesenteric arteries than that of the kidney; therefore all further experiments were performed only in the mRNA samples isolated from the mesenteric arteries of Untrt and IRW‐treated groups.

### 3.4 Target gene validation

Functional analysis showed that DE genes in the mesenteric artery modulated different cardiovascular functions. Six most interesting DE genes, ACE‐2, ABCB‐1, IRF‐8, CDH‐1 ICAM‐1, and VCAM‐1, were selected for further analysis using qRT‐PCR. According to qPCR analysis, expression of four target genes, ACE‐2, ABCB‐1, IRF‐8, and CDH‐1, were significantly upregulated, while expression of two target genes, ICAM‐1 and VCAM‐1, were significantly downregulated in the mesenteric artery of IRW group (Table 1 *)*. The potential roles of these genes in modulating cardiovascular disease condition and the impact of IRW treatment are presented in Table 1. These six genes have significant roles in all of these pathways and significant fold change (increase or decrease) was observed in the mesenteric artery tissues, qPCR results are in alignment with the RNAseq data (Table 1).

## 4 Discussion

The relationship between diet and hypertension has been largely recognized by various epidemiological studies 8, 31, 32. Recent studies have characterized various food bioactive compounds that may be responsible for the treatment and management of hypertension and associated cardiovascular disorders 32, 33, 34, 35. Most of these compounds are plant secondary metabolites such as polyphenols and flavonoids 36, 37; food protein derived peptides with blood pressure lowering, cholesterol lowering, anti‐thrombotic, and anti‐oxidant activities have been proven beneficial against CVDs 38, 39, 40. These bioactive compounds can activate signaling pathways and then regulate the expression of specific genes and consequently their translation into the corresponding proteins 41. There are several studies performed to identify the gene expression changes after treatment of bioactive peptides, using DNA microarray or quantitative real‐time PCR (qRT‐PCR) techniques 22, 23. DNA microarray analysis have suggested that IPP and VPP could significantly increase the expression of eNOS, connexin 40, and cyclooxygenase‐1 (COX‐1) genes, whereas decreased the expression of nuclear factor kappa B subunit (NF‐κB) and peroxisome proliferator activator receptor gamma (PPARγ) genes in SHRs 10. Using RT‐PCR analysis, flavonoids, and phenolic compounds from lingonberry and cranberry have been shown to reduce the expressions of ACE, cyclooxygenase‐2 (COX‐2), monocyte chemoattractant protein‐1 (MCP‐1), and P‐selectin genes and exhibit anti‐inflammatory and anti‐thrombotic effect in SHRs 42. Another study showed that peanut polyphenols could exert hypolipidemic effects by increasing the expression of fatty acid synthase, sterol receptor element‐binding protein‐1c, acetyl‐CoA carboxylase (ACC1), and PPARγ genes in Wistar rats 43, 44. In this study, we used the nutragenomics 45 based approach to study the effect of oral administration of a bioactive peptide on gene expression profile of vascular tissues through transcriptome analysis performed by RNA‐seq, a high throughput analysis that could delineate the link between phenotype and information encoded in the genome, which could be used to understand the molecular mechanism of an active food compound. Our study showed that egg protein derived bioactive peptide IRW altered the gene expression in the vascular tissues in adult male SHRs; and 11 890 genes are similar in both kidney and mesenteric arteries but they differentially expressed more in mesenteric arteries than kidney. Functional analysis revealed that DE genes identified from mesenteric arteries have known modulatory roles in various cellular functions, whereas DE genes in kidney have no significant impact at functional level, suggesting a pronounced effect of IRW treatment on mesenteric arteries.

Most reported antihypertensive peptides target at the RAS at the level of ACE inhibition or Ang II receptor blockade. For example, a study by Yu et al. reported that administration of ACE inhibitory peptide, RVPSL, for 4 weeks significantly reduced the mRNA expressions of renin, ACE, and AT_1_ receptor in kidney 23. However, specific modulatory effects of bioactive peptides on ACE‐2 remain largely unknown. Our study showed that IRW treatment significantly increased the gene expression of ACE‐2 without affecting RAS components. Unlike ACE, ACE‐2 functions as a carboxy‐peptidase, cleaving a single residue from the vasoconstrictor Ang II to generate a vasodilatory peptide angiotensin 1–7 (Ang_1‐7_). A G‐protein‐coupled receptor ‐Mas acts as the Ang_1‐7_ receptor and initiates a counter‐regulatory role by opposing Ang‐II induced vasoconstriction. Thus ACE‐2 contributes to an alternate arm of the RAS pathway, which counterbalances the effects of the classic RAS pathway through degradation of Ang II 46, 47. The reduction of plasma angiotensin‐II level after IRW treatment observed in our previous study 18 could be mediated, at least partially, through increased expression of ACE‐2, as revealed in the present study. A recent study by Ehlers et al. concluded that the milk‐derived ACE inhibitory peptide IPP might exert additional antihypertensive effects through activation of the ACE‐2‐Ang_1‐7_‐Mas axis of RAS system, although expression of ACE‐2 was not determined 7. ACE‐2 is a newly identified arm of RAS pathway; upon activation it can play a vasodilatory role to reduce high blood pressure 46, 47. Therefore increasing the expression of ACE‐2 could mediate the antihypertensive effect through the degradation of vasoconstrictory compound Ang II. Thus, our study documented for the first time that a food protein‐derived ACE inhibitory peptide IRW could contribute to its antihypertensive effect through a new mechanism: increased gene expression of ACE‐2.

Our present study also revealed that IRW reduced gene expression of ICAM‐1 and VCAM‐1 in the mesenteric arteries. The reduced gene expression indicates the suppression of protein upregulation at the transcriptional level, which correlates with the reduced ICAM‐1 and VCAM‐1, observed in our previous in vivo study 18. Cellular activation of the proinflammatory transcription factor NF‐κB causes nuclear translocation of p65‐p50 dimers and thus enhances expression of genes whose protein products (such as ICAM‐1 and VCAM‐1) mediate leukocyte recruitment under inflammation and consequently contribute toward vascular inflammation and atherosclerosis 48, 49. We also demonstrated that IRW treatment could inhibit the translocation of both p65 and p50 in endothelial cells 17, which is in agreement with the present study showing downregulation of ICAM‐1 and VCAM‐1 gene expression in the IRW group.

**Figure 4:**
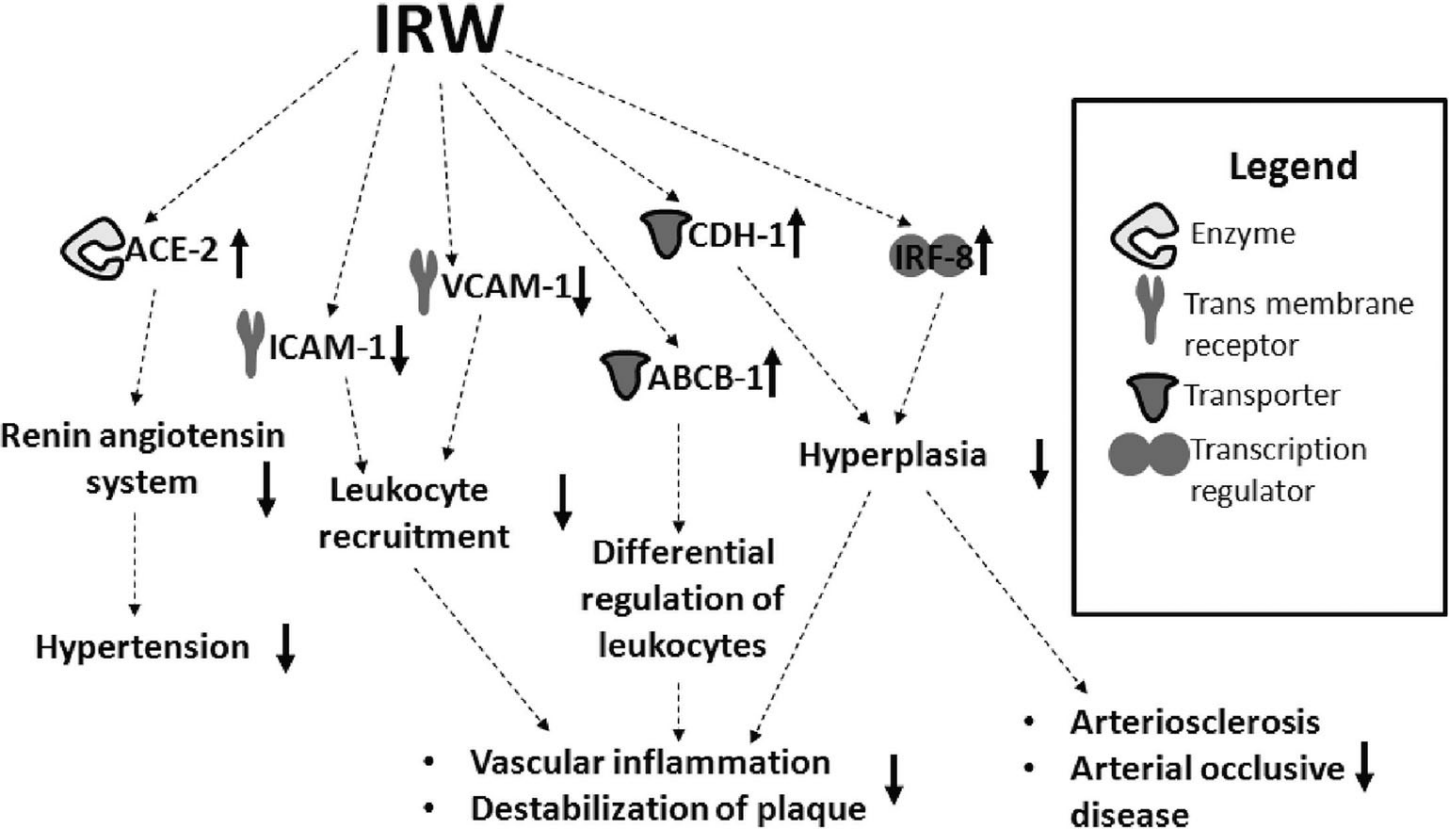
A schematic representation of the effect of IRW on various gene expressions and how it can modulate cardiovascular disease function. The DE genes identified from the mesenteric artery of the IRW treated group modulate various cardiovascular functions. IRW treatment increases the gene expression of angiotensin converting enzyme‐2 (ACE‐2), which can break down vasoconstrictor angiotensin‐II and reduce the activity of renin angiotensin system (RAS), leading to reduced blood pressure. Furthermore, IRW treatment can reduce the gene expression of intercellular adhesion molecule‐1 (ICAM‐1) and vascular cell adhesion molecule‐1 (VCAM‐1) that decrease the leukocyte recruitment in the vasculature and reduce the risk of vascular inflammation, destabilizing atherosclerotic plaque. Similarly, IRW treatment increases the gene expression of ATP‐binding cassette, subfamily B (ABCB‐1), reducing vascular inflammation through differential regulation of leukocytes. On the other hand, IRW treatment increases the expression of E‐Cadherin (CDH‐1) and interferon regulatory factor 8 (IRF‐8) that reduces the hyperplasia of vasculature and the risk of arteriosclerosis and associated cardiovascular disorder.

Another two DE genes are the transporters, CDH‐1 and ABCB‐1. Expression of these two genes was significantly increased after IRW treatment. As a member of ABC (ATP‐binding cassette) proteins, ABCB‐1's primary role is to provide a first line of defense at the cellular level by inhibiting entry of potentially harmful compounds into the cell and excreting toxic metabolites out of the cell 50. Overexpression of ABCB‐1 also controls the production of leukocyte and thus can control the inflammatory response 51. It was also reported that overexpression of ABCB‐1 might affect the lipid homeostasis and could increase the reverse transport of cholesterol 52. Therefore upregulation of ABCB‐1 could modulate vascular inflammation via differential regulation of leukocytes and could be helpful in destabilization of atherosclerotic plaque through reverse transport of cholesterol 53. Some studies suggest that polymorphisms in this transporter gene might affect individual's susceptibility to ischemic heart disease and atherosclerosis, but the results are still inconclusive 54, 55, 56. Moreover, apart from CVDs, increased expression of ABCB‐1 gene can also reduce the risk of colitis and inflammatory bowel disease 57, suggesting a potential role of IRW in treating several other disease conditions in addition to CVDs. Another transporter gene, CDH‐1 expression was also significantly upregulated by IRW treatment. CDH‐1 is a protein belongs to cadherin family whose main function is to help neighboring cells stick to one another (cell adhesion) to form organized tissues 58. Upregulation of CDH‐1 could inhibit the hyperplasia of endothelial and vascular smooth muscle cells 59, 60. Similarly, another target gene IRF‐8 was significantly upregulated by IRW, which could also inhibit the hyperplasia of the vascular cells 61. These results suggest that IRW treatment can reduce vascular inflammation as well as the hyperplasia of the vascular smooth muscle cells, both of which predispose to arteriosclerosis, a complication of long standing hypertension 62, 63. Increased inflammation and oxidative stress can also lead to fibrosis and remodeling in vascular tissues. Our previous study demonstrated that IRW treatment significantly attenuated type I collagen levels 18, suggesting a reduction in hypertension induced tissue remodeling. Results from the current study are in alignment with our previous findings as the upregulation of CDH‐1 and IRF‐8 genes inhibits hyperplasia, which may leads to the alteration of tissue fibrosis and vascular remodeling. Additionally, feeding IRW for a period of 18 days did not affect body and organ weights (data not shown), suggesting it is likely safe; further phase‐I clinical trial should be performed to validate its safety. Schematic representations of IRW treatment on gene expressions are illustrated in Fig. 4.

In conclusion, IRW increased expression of genes for anti‐hypertensive and anti‐inflammatory mediators in vasculature concomitant to a downregulation in proinflammatory genes in SHRs. These results may explain the mechanisms underlying the anti‐hypertensive, anti‐inflammatory, and improved endothelial functions observed upon IRW treatment in vivo. We believe that this is the first study to observe a food‐derived bioactive peptide upregulate expression of ACE‐2 gene, a vasodilator and anti‐inflammatory molecule that may open up novel therapeutic opportunities for the management of vascular and associated inflammatory diseases. Given the oral route of administration, IRW may indeed be well suited for translation as a nutraceutical targeting a number of cardiovascular pathologies. As both RAS and inflammatory pathways affect diverse tissues and organs, a systematic approach to determine IRW's effects on various physiological and pathological processes is necessary. Indeed our transcriptomics study provides novel evidence supporting the role of IRW in modulating the mechanisms underlying conditions such as inflammatory, metabolic and cardiovascular diseases.

*The authors have declared no conflict of interest*.

## Supporting information

As a service to our authors and readers, this journal provides supporting information supplied by the authors. Such materials are peer reviewed and may be re‐organized for online delivery, but are not copy‐edited or typeset. Technical support issues arising from supporting information (other than missing files) should be addressed to the authors.

Table S1. PCR‐primer pair for selective gene

Table S2.

Table S3.
