# Supplementary material for: Egg ovotransferrin‐derived ACE inhibitory peptide IRW increases ACE2 but decreases proinflammatory genes expression in mesenteric artery of spontaneously hypertensive rats
Source: Mol Nutr Food Res. 2015 Jun 26;59(9):1735–44. doi: 10.1002/mnfr.201500050 (PMC5034750; doi:10.1002/mnfr.201500050)
Supplement: Supplementary file 1 — Table S1. PCR‐primer pair for selective gene [file MNFR-59-1735-s001.docx]

**Supplementary Table 1: PCR-primer pair for selective gene**

| **Gene Name** | **Primer Pair** | **Temperature** | **Reference** |
| --- | --- | --- | --- |
| ACE-2 (Angiotensin Converting Enzyme2) | Forward Primer GCTAAACATGATGGCCCACT  Reverse Primer CCCACAGTCGAATTCCTGTT | 64^o^C | Designed and verified in this study |
| ABCB-1 (ATP-Binding Cassette, sub-family B-1) | Forward Primer CGTTGCCTACATCCAGGTTT  Reverse Primer TGGAGACGTCATCTGTGAGC | 62^o^C | Designed and verified in this study |
| ICAM-1 (Intercellular Adhesion Molecule-1) | Forward Primer GAGTCTCCCAGCACCAGCAT  Reverse Primer GTGCCTACCCTCCCACAACA | 64^o^C | Designed and verified in this study |
| VCAM-1 (Vascular cell Adhesion Molecule-1) | Forward Primer GAGTCTCCCAGCACCAGCAT  Reverse Primer GTGCCTACCCTCCCACAACA | 60^o^C | Designed and verified in this study |
| IRF-8 (Interferon Regulatory Factor-8) | Forward Primer ACGCAGGCAAGCAAGACTACA  Reverse Primer AATCCGGGCTCTTGTTCAGA | 62^o^C | Designed and verified in this study |
| E-Cadherin-1 (CDH1) | Forward Primer GGGTTGTCTCAGCCAATGTT  Reverse Primer CACCAACACACCCAGCATAG | 64^o^C | Designed and verified in this study |
| β-actin | Forward Primer CTAGGCACCAGGGCGTAATG  Reverse Primer CCACACGGAGCTCGTTGTAG | 60-64^o^C | [64] |
